# Novel Method for Neuronal Nanosurgical Connection

**DOI:** 10.1038/srep20529

**Published:** 2016-02-05

**Authors:** Nir Katchinskiy, Helly R. Goez, Indrani Dutta, Roseline Godbout, Abdulhakem Y. Elezzabi

**Affiliations:** 1 Department of Electrical and Computer Engineering, University of Alberta, Edmonton, AB, Canada; 2 Division of Pediatric Neurology, Department of Pediatrics, Faculty of Medicine and Dentistry, University of Alberta, Edmonton, AB, Canada; 3 Department of Oncology, University of Alberta, Cross Cancer Institute, Edmonton, AB, Canada

## Abstract

Neuronal injury may cause an irreversible damage to cellular, organ and organism function. While preventing neural injury is ideal, it is not always possible. There are multiple etiologies for neuronal injury including trauma, infection, inflammation, immune mediated disorders, toxins and hereditary conditions. We describe a novel laser application, utilizing femtosecond laser pulses, in order to connect neuronal axon to neuronal soma. We were able to maintain cellular viability, and demonstrate that this technique is universal as it is applicable to multiple cell types and media.

Neurons are cells within the nervous system that process and transfer information through electrical impulses and chemical signals^1^,^2^. This type of communication between neurons occurs in a specific interphase called a synapse^2^. The Central Nervous System (CNS) which includes the brain and the spinal cord, as well as the Peripheral Nervous System (PNS) operate in a similar pattern utilizing a neuronal synaptic network as a means of intercellular communication. It is imperative to have intact neuronal circuits in order to maintain normal organ and organism function. The typical neuronal structure includes cell body (soma), dendrites, and an axon. Dendrites are structures arising from the cell body branching multiple times. An axon is a cellular extension arising from the cell body which can traverse a long distance in human beings and in other species. The axon may branch multiple times and connect to multiple cells prior to termination. There is an average of 300 trillion synapses in the adult brain^3^.

Neural communication is usually transferred through one neuron’s axon to a dendrite or cell body of another neuron. However, there are other connection options based on different neuronal cell structures: for example, axon to axon, dendrite to dendrite, etc^4^. The cell membrane of the soma and the axon is electrically conducting through activation of voltage-gated ion channels^4^. These calcium, sodium, potassium, and chloride ion channels generate the electrical signals propagated in the axons.

Neurons are highly specialized cells that can no longer divide. Neurogenesis is very limited in adulthood; hence, nerve injury has a major impact on the ability to maintain normal function. There are a few recognized patterns to peripheral nerve damage post injury: Wallerian degeneration, segmental demyelination and axonal degeneration^5^,^6^,^7^,^8^,^9^,^10^. Wallerian degeneration develops after transection of the nerve and injury to the axon and its myelin sheath, where distal to the transection both the axon and the myelin will degenerate. A conduction block is observed one week after the injury. Regrowth is possible depending upon preservation of the basement membrane of the cells that are producing myelin (Schwann cells) and approximation of the nerve ends^5^,^6^. This type of damage may cause atrophy of the muscle innervated by the damaged neuron. Segmental demyelination is the result of damage restricted to the myelin sheath. As the axon is preserved, no end point muscle degeneration is expected or observed^6^,^7^. Axonal degeneration is caused by damage to the neural cell body resulting in distal death of the axon. Muscle atrophy will develop unless re-innervation occurs from adjacent nerves, however recovery may be only partial^8^,^9^,^10^. Experimental work on nerve injury repair has been carried out in the last decade and a few approaches have been investigated. These techniques included: bridging a gap by utilizing growth permissive matrix placed across the site of injury to allow axonal growth^11^, creating new network via stem cell therapy^11^, providing neurotropic support in order to stimulate sprouting of spared axons or enable regeneration of injured axons^11^,^12^. Alternative techniques attempted to overcome myelin associated growth inhibitors^11^,^12^, and scar-associated growth inhibitors^11^,^12^. Molecular protection of cells was used to avoid from the cascade of biochemical events that lead to cell death post injury^12^. These investigational treatments were encouraging but had partial success in animal models.

It is of paramount importance to develop a precise means of selectively connecting specific axons to neuron cell body. Such a leap in scientific method will open up doors to unparalleled research frontiers in neurology, cell biology, biochemistry, and electrophysiology. Connecting neurons, before or right after injury, enables the preservation of the viability of the neural network, thereby allowing complex pathophysiological processes, such as neurogenesis, Wallerian degeneration, segmental demyelination, and axonal degeneration to be further understood. Understanding the complex pathophysiological processes and the time frame available in order to prevent conductivity block and axonal death makes it necessary to develop techniques that enable the connection of nerve ends as soon as possible post injury, and maintain the viability of a healthy neural network. We describe a novel laser application to physically reattach severed neurons right after injury. This method may potentially allow further prevention of a conductivity block. Moreover, it may trigger studies questioning the hypothesis whether physical attachment and approximation of the nerve ends will stimulate recovery.

To date, a method to connect neuron ends does not exist. Assessment of axonal growth and regeneration is currently performed via either immunolabeling, where specific proteins that are involved in known regeneration pathways are labeled and monitored or via anterograde and retrograde tracing to visually trace neural connections from their termination/source to their source/termination. These imaging methods are utilized to trace the neuronal projections from one location to various targets in the nervous system, and it allows researchers to study the natural process of axonal regeneration. However, the above mentioned techniques are limited to studying only the natural healing processes of neurons. Thus, control on selection and isolation of neurons, in order to study regeneration of specific neurons, is not available. Knowledge gained from such studies will allow researchers to develop new therapies for, currently, irreversible neuronal injuries and diseases.

A prime candidate method for connecting specific neurons that fulfills such key applications is femtosecond laser pulse technology. This versatile technology has been utilized for very precise cell manipulation, such as optoporation, cell nanosurgery, cell isolation, and embryo transfection^13^,^14^. Removal or ionization of material is confined to less than a diffraction limited spot size, with no damage to surrounding material. Femtosecond laser pulses have also been used as a tool to study neuron regeneration by severing neurons and axons^15^. This method allows creating of precise injury that enables the studies on axonal injury and regeneration at the single cell level^15^. More recently, it was demonstrated for the first time that this technology can be used to “reverse” cell cutting or isolation, by performing cell-cell attachment^16^. However, physical connection of single neurons has not been performed thus far.

In this communication we present a method for neuron connection, using femtosecond laser pulses. By physically connecting single axons and neurons right after injury, it will allow researchers to develop new methods of studying the effects of neuron connection on neuronal regeneration, progression of Wallerian degeneration, and the existence of cellular communication, to further our understandings of these phenomena. This effective neuronal connection method should allow the user to select single cells for isolation, connection, and cutting. The technique is shown to be universal and applicable to multiple cell types and their media.

We developed a novel neuron connection method using ultrashort femtosecond laser pulses (illustrated in Fig. 1a). Precise tuning of the laser parameters allowed us to induce a process called hemifusion at the contact point of two phospholipid membranes (illustration of the contact point is seen in Fig. 1b). To achieve neuron connection, the laser intensity and aiming accuracy required are 1.7(±0.08)×10^12 ^W/cm^2^, and ±0.5 μm, respectively, within the membranes hemifusion location. Exposure to near infrared femtosecond laser pulses induces molecular rearrangement of the phospholipid bilayers via multiphoton and avalanche ionization processes. The high electron and ion density at the laser beam focal point leads to an ultrafast reversible destabilization of the phospholipid molecules. Since the membrane’s exterior surface is permeable to both photo-induced ions and electrons, these can cross over to the central nonpolar region of the phospholipid bilayer and break the bonds of the fatty acid tails, as illustrated in Fig. 1c. At the end of this destabilization process, the ionized phospholipid molecules seek equilibrium state, and form new bonds with nearby ions, as seen in Fig. 1d. Only the phospholipid molecules that are located at the cell membrane contact point cross-link with phospholipid molecules of the adjacent cell membrane. The cross-linking process leads to the formation of a single, shared, phospholipid bilayer (i.e. hemifused membrane), which is the underlying mechanism that takes place in this neuron connection method and provides a strong attachment.

To demonstrate our neuron connection method, we show that this technique can be used on any number and types of neurons by its implementation on two neuron types: P19, and Neuro2A. Neurons were grown in culture, and suspended in DMEM solution right before connection (see online methods). Selected neurons for connection were identified, isolated and brought into contact using an optical tweezer such that the protruding axon of one neuron touched another neuron’s soma. In order to ensure that the neurons do not naturally stick to each other, the cells were left touching for a period of time, and then pulled apart by the optical tweezer. The neurons did not show any signs of natural connectivity. The neurons were brought into contact once more, and then femtosecond laser pulses were delivered to the axon and cell soma connection point (see methods) in order to induce an attachment. To validate that a connection was achieved, one of the neurons was moved inside the suspension dish using optical tweezer (see methods), and it was found that all neurons followed a corresponding path, twisted, and rotated as a single entity, without showing any sign of detachment. Figure 2a depicts the attachment of two Neuro2A cells (see methods), where the axon of cell (i) is attached to the soma of (ii).

Connection of single neuron to multiple neurons is a fundamental requirement for assembling a chain of neurons, and to maintain neuronal connectivity and continuity. As shown in Fig. 2b, two axons from Neuro2A (i) were attached to Neuro2A (ii) and (iii). The cells are shown, in Fig. 2c, after being moved and oriented approximately 30° relative to their original orientation.

Figure 2d–f depict a femtosecond laser induced connection of P19 axon to P19 soma (see methods). Here, connection of groups of targeted neurons is demonstrated. Two groups of four P19 cells were identified, as shown in Fig. 2d. The axon of neuron (i) came in contact and was connected with (ii), using femtosecond laser pulses. In Fig. 2e the cells are shown right after attachment, and in Fig. 2f the cells are shown after being pulled and rotated using the optical tweezer.

Several groups of neurons were attached in order to demonstrate the proposed neuron connection method. One to two 15 ms pulse trains (i.e. 1.2 × 10^6^ pulses) were necessary to achieve attachment, with 90% success rate. Throughout our observations and rigorous manipulations, the cells remained viable and firmly attached without showing signs of deterioration in attachment strength, validating long term viability and attachment prospects. Moreover, previously reported cell-cell attachment method^16^, and long term viability experiments^17^,^18^ also confirm that the laser parameters used for this method fall within a safe range for preserving cell viability and attachment. We envisage that femtosecond laser-induced neuronal nanosurgical connection method can potentially provide a scientific leap that will open up new frontiers in the studies of the effects of connecting neurons, right before or after injury. The preservation of the viability of the neural network will allow researchers to study new complex pathophysiological processes, such as neurogenesis, Wallerian degeneration, segmental demyelination, and axonal degeneration. This will allow further development of new therapies for neuronal injuries and disease.

## Materials and Methods

### Cell Cultures

P19 mouse teratocarcinoma cells^19^ were cultured in Dulbecco’s Modified Eagle’s Medium (DMEM) supplemented with 7.5% bovine serum and 2.5% fetal calf serum. For neuronal differentiation, P19 cells were cultured in petri dishes (to allow formation of embryoid bodies) at a density of 10^5^ cells/ml in the presence of 1 μM all-trans-retinoic acid (RA) (Sigma R2625) (Day 0)^20^. On Day 2, the medium was replaced with fresh DMEM supplemented with serum and 1 μM RA. On Day 4, the embryoid bodies were trypsinizsed and broken down into single cells. These single cells were plated on coverslips and cultured in DMEM plus 10% fetal calf serum. On Day 6, the cells were treated with 5 μg/ml Ara-C (Sigma C1768) in order to remove any remaining proliferating cells. The cells completed neuronal differentiation by Day 8, at which stage long neurite projections could be observed. Neuro2A (mouse neuroblastoma cells) were cultured in DMEM supplemented with 10% fetal calf serum, penicillin (100 U/ml) and streptomycin (100 μg/ml). At confluence, cells were trypsinized and plated on coverslips.

### Setup Characteristics

The neuron connection was achieved by using sub-10 femtosecond laser pulses, with 800 nm central wavelength that was delivered from a Ti:Sapphire laser oscillator at a repetition rate of 80 MHz. The near infrared pulses were coupled to an upright Nikon Eclipse 80i optical microscope and directed towards the cells. A 60× (numerical aperture (NA) = 1) water immersion microscope objective was used, in order to focus the laser pulses and to image the cells. A high NA microscope objective is required to achieve high-resolution imaging and to focus the beam to an effective spot size of ~600 nm. At the focal spot, the optimum laser pulse train average power, energy, and intensity are: 200 mW, 2.5 nJ/pulse, and 1.71 × 10^12 ^W/cm^2^, respectively. Ideal irradiation time is 15 ms pulse train (i.e. 1.2 × 10^6 ^pulses). The neuron connection experiments were imaged and recorded in real-time using a color charge-coupled device (CCD) camera. The images used in this communication were taken from the video recordings after the completion of the experiment.

### Experimental Procedure

P19 and Neuro2A cells were placed inside a glass dish with DMEM solution and mounted on a motorized x-y-z nano-translation stage for precise movement control of the cell culture. Trypsin solution was added to each dish in order to release the neurons from the bottom of the plate. After 10 minutes, the dish was shaken by hand in order to suspend the neurons in the solution. Neurons with identifiable axons and that are not attached to large groups have been identified and selected for connection. The selected neurons were brought into contact using an optical tweezer, such that an axon of one neuron touches the soma of the other neuron. The optical tweezer was made collinear with the femtosecond laser pulse train. Once the desired contact region between the axon and cell soma was identified, it was precisely targeted by the femtosecond laser pulse train for a duration of 15 ms. The mechanical integrity of the connected neurons was then assessed using an optical tweezer. This was performed by trapping one of the neurons to the optical tweezer’s focal spot, and moving the trapped cell(s) in various paths. In order to verify that proper attachment was obtained, three criteria were assessed: (1) no detachment due to twisting, and drag forces (2) movement of all cells due to trapping of any cell in the group, and (3) movement of all cells as a single unit. We confirmed the physical cellular attachment by following the translation of the trapped cell as an integral unit together with the other cells without detaching from each other. In order to verify that the connected neurons did not move only due to their proximity to the optical trap, groups of four neurons were attached. Using this technique we were able to ensure that the neurons are located far enough from the optical trapping spot, where the attraction forces to the laser tweezers are too weak to pull an individual cell.

## Additional Information

**How to cite this article**: Katchinskiy, N. *et al.* Novel Method for Neuronal Nanosurgical Connection. *Sci. Rep.* **6**, 20529; doi: 10.1038/srep20529 (2016).

## Figures and Tables

**Figure 1:**
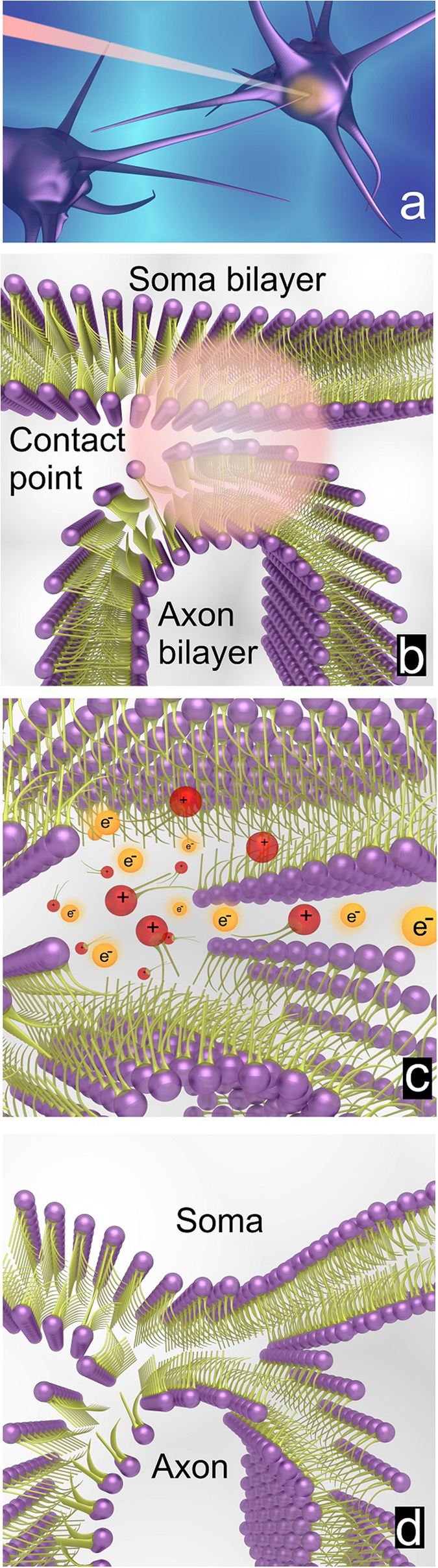
(**a**) An illustration of how a femtosecond laser pulse is delivered to the target point between an axon and a neuronal soma. (**b**) An illustration of the phospholipid bilayers of the neuron soma and axon. Note that the attachment region, where the phospholipid layers are attaching, is designated with a circular spot. This does not represent the laser focal spot. (**c**) The laser pulse high intensity causes a reversible destabilization of both phospholipid layers. A depiction of the femtosecond laser pulse induced axon-soma attachment. Here, the generated free ions (shown in red) and free electrons (shown in orange) cross the center nonpolar region and break bonds between the fatty acid hydrophobic tails. (**d**) The relaxation process results in the formation of new stable bonds and formation of singular, hemifused, cell membrane only at the targeted connection point.

**Figure 2:**
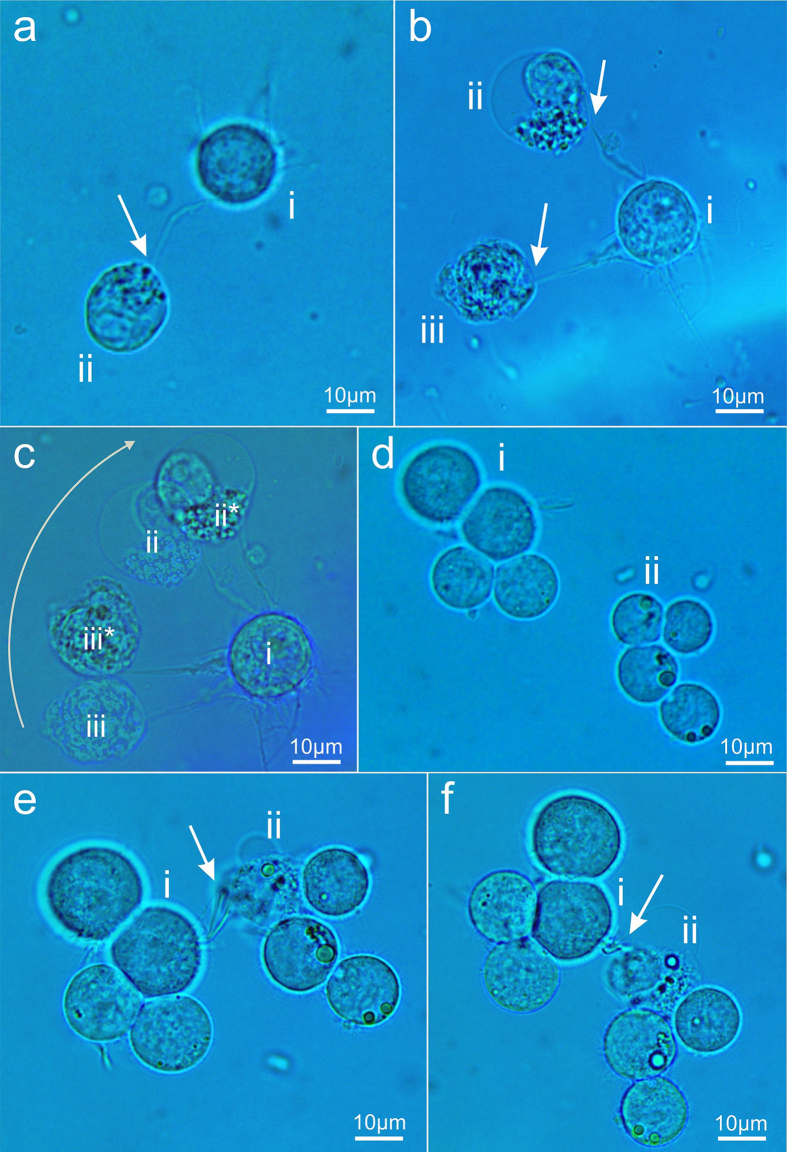
The arrows point to the location of neuron connection using femtosecond laser pulses. (**a**) A single Neuro2A’s (**i**) axon is connected to the soma of Neuro2A (**ii**). (**b**) Two axons of Neuro2A (**i**) are connected using to the soma of Neuro2A cells (**ii**) and (**iii**). (**c**) Overlap image of the original Neuro2A cells (**i**,**ii**), and (**iii**) compared to Neuro2A cells (**i**), (ii*), and (iii*) after being rotated and moved by an optical tweezers to examine the integrity of the axons’ connectivity. (**d**) Two groups of four P19 neurons are identified. (**e**) The axon of P19 (**i**) is brought into contact with the soma of P19 (**ii**), and are connected as shown by the arrow tip. Note that there is another unconnected axon nearby which was left detached (**f**) The two groups of P19 cells are rotated and moved by optical tweezers to examine the integrity of the axons’ connectivity.
